# Erie County Medical Society

**Published:** 1911-11

**Authors:** 


### Erie County Medical Society

**Received too late for publication in proper place**

The regular quarterly meeting of the Medical Society of the County of Erie was held, on Monday evening, October 16, 1911, in the Buffalo Library Building, with President McClure presiding.

Minutes of the previous meeting and the Council meetings were approved.

New members were elected as follows:

Louis A. Kaiser, Alfred F. Luhr, John G. Chadwick, James C. Haley, R. Montfort Schley, George F. Harris, Chester C. Cott, all of Buffalo, N. Y., R. H. Wilcox, Tonawanda, N. Y., and Allen L. Haenszel, Ebenezer, N. Y.

Dr. George L. Brown, Chairman of the Board of Censors, submitted a detailed report of the work of the Board in which, among other things, he gave an account of the various prosecutions since the last meeting, the important ones being as follows:

For the illegal practice of medicine, a fine of twenty-five dollars was imposed and collected from each of the following:

Fannie Cirese, Joseph Stein, Agnes Wrzesinska, Anna Miller. The first named has left the city.

Drs. A. E. Collins, Matthew Willoughby, Frank B. Voght and Charles Monroe Manges were reported as being under bail for alleged criminal practices.

Several other flagrant violators of the medical law were driven out of town, the most important of which was a Mr. Mereroff, proprietor of the Porter Medical Company which company has been doing business in Buffalo and many other cities for a number of years.

Mr. Mereroff gave a bond for his appearance, but his case has not yet come to trial. The Buffalo offices of the company have, however, been closed since last spring.

Dr. T. H. McKee, Chairman of a Special Committee appointed for the purpose of obtaining the records of public officials on sanitary and health matters, made a report in which he stated that the large number of physicians of Buffalo and Erie County ought to wield a powerful influence for good in this behalf, if their influence was exerted in a systematic manner. Furthermore, if the public officials knew that their actions were being watched by the medical fraternity, the results, in many instances, would be different.

On his motion, an appropriation of one hundred dollars was set aside to be used for this purpose during the coming year.

On motion of Dr. McKee, the following resolution was adopted:

"Whereas, a concerted attempt is being made to undermine the authority and efficiency of the Health Department and to subject the community to the risk of smallpox epidemics by abrogating that section of the ordinances which makes vaccination compulsory, be it

*Resolved,* That the members of the Erie County Medical Society hereby register a vigorous protest against any such change in the law; and further

*Resolved,* That we heartily endorse the attitude of the Health Commissioner, and especially commend him for the stand he has taken in this matter, as well as in relation to pure food regulations, and that a copy of these resolutions be forwarded to the Aldermen, the Councilmen and the Mayor."

Also, on motion of Dr. McKee, the Delegates to the Medical Society of the State of New York, were instructed to urge, at the next meeting of said Society, the desirability of proper teaching on the subjects of smallpox and vaccination, in the various medical colleges, it having been demonstrated that many graduates are absolutely uninformed on this subject.

